# Association between periodontitis and ossification of the posterior longitudinal ligament: a case report

**DOI:** 10.1186/s13256-021-03142-4

**Published:** 2022-06-13

**Authors:** Michiyo Tsuru, Takayoshi Oosio, Teruyo Higashi, Kensei Nagata, Kanichiro Wada, Yasuyuki Ishibashi

**Affiliations:** 1 Clinical Proteomics and Gene Therapy Laboratory, Kurume University, Fukuoka, Japan; 2 Japan Regenerative Medicine Center Co., Ltd, Cell Medical Team Japan Med. Corp., Dental Team Japan Med. Corp., 3-30-15 Hakata Ekimae, Hakata, Fukuoka 812-0011 Japan; 3 Oosio Dental Clinic, Takaikekubo 43-3 Kokufucho Ido, Tokushima, 779-3118 Japan; 4 Morino Clinic, 2 -71 Showacho, Tokushima, Tokushima 770-0942 Japan; 5 Department of Orthopaedic Surgery, Kurume University School of Medicine, 67 Asahi-machi, Kurume City, Fukuoka 830-0011 Japan; 6 Department of Orthopaedic Surgery, Hirosaki University Graduate School of Medicine, Aomori, 036-8562 Japan

**Keywords:** Cytokines, Zinc, Ligaments, Ossification of the posterior longitudinal ligament, Periapical periodontitis

## Abstract

**Introduction:**

Ossification of the posterior longitudinal ligament in the spinal ligament compresses the spinal cord, causing various spinal nerve diseases. The ligament tissue is an important connective tissue in the joints, teeth, and spine, which, when torn and damaged, reduces the range of movement.

**Case presentation:**

We report the treatment of periapical periodontitis and tooth preservation in a 41-year-old Japanese woman previously diagnosed with ossification of the posterior longitudinal ligament. She presented with widespread pain from the paranasal sinuses to the head caused by the onset of periapical periodontitis of the upper front tooth.

**Discussion:**

The patient received an oral zinc supplement, which resolved periapical periodontitis over a 2-year follow-up period.

**Conclusion:**

The findings from this case imply that, when patients with ossification of the posterior longitudinal ligament develop periapical periodontitis, they should be tested for zinc deficiency.

## Introduction

Prior to this study, we induced the growth of ligament-like tissue from mesenchymal stem cells [1] and created a mouse model for ossification of the posterior longitudinal ligament (OPLL) for the study of ligament diseases [2]. In transgenic mice, ligament-like tissue derived from mesenchymal stem cells was found to be strongly expressed in the spinal ligament, anterior cruciate ligament of the knee joint, and periodontal ligament. Ligaments are connective tissues that connect hard tissues, including the joints, teeth, and spine. Tears and damage to the ligament tissue can reduce the range of motion of the affected joint. The periodontal ligament, also known as the periodontal membrane, strongly attaches teeth to the alveolar bone. It also receives tactile and sensory stimuli, conveys signals to the brain, and regulates the bite force. The spinal ligament tissue supports the cervical spine (7 vertebrae), thoracic spine (12 vertebrae), and lumbar spine (5 vertebrae). The tough anterior longitudinal ligament adheres to the anterior surface of the vertebral bodies from the skull base to the sacrum, while the posterior longitudinal ligament adheres to the posterior surface of the vertebral bodies from the clivus to the inside of the sacral canal. The ligamentum flavum is present in the posterior portion of the vertebral arch inside the spinal canal, whereas the supraspinatus and interspinous ligaments are located at the back of the spinal column. These ligaments provide strong support to the spinal column and facilitate mobility.

The vertebrae were anchored by longitudinally extending the ligament tissue. OPLL is an incurable disease of unknown cause in which the spinal ligament ossifies, resulting in limited range of motion and reduced function of the extremities. A previous proteomic study [2] identified a partial defect in the chemokine C-X-C motif ligand 7 (CXCL7) and low serum levels among patients. OPLL is classified into continuous, segmental, mixed, and circumscribed subtypes. Serum CXCL7 levels, measured by enzyme-linked immunosorbent assay, were reduced in patients with continuous OPLL compared with those with mixed OPLL. In healthy subjects, serum CXCL7 levels were > 11 ng/mL; however, patients with the circumscribed and segmental types of OPLL showed levels of approximately 4 ng/mL; patients with the mixed type of OPLL showed levels of approximately 3.3 ng/mL, and patients with the severe continuous type showed levels of < 2 ng/mL.

**Fig. 1:**
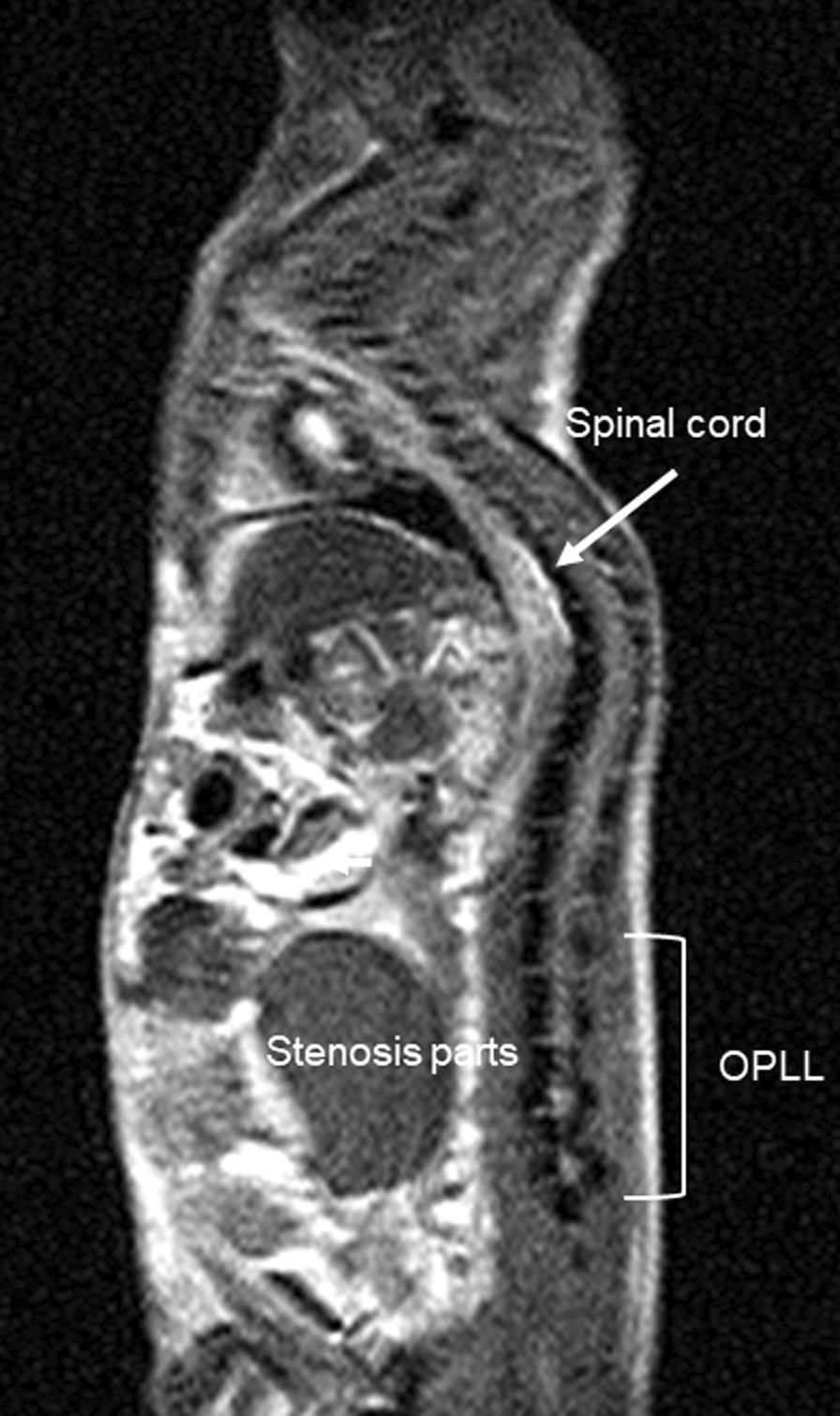
Magnetic resonance imaging showing ossification of the posterior longitudinal ligament in a mouse model of thoracic-to-lumbar spine measurement of bone morphology indicates that these genetically engineered mice are in a state of dynamic bone ossification

Previous studies have created mesenchymal stem cell-derived ligament tissue models for the *in vitro* demonstration of OPLL [1, 2]. Based on the results of a genome array of the tissue model, the ligament tissue was considered to be associated with zinc [1]. We further identified the deficiency of a portion of CXCL7 in patients with OPLL and succeeded in creating a mouse model for studying OPLL (Fig. 1) [2]. In addition to the findings of these studies, the present case findings would be a valuable guide the treatment of ligament disease. The patient was informed that data concerning the case would be submitted for publication, and she provided written informed consent.

## Case presentation

**Fig. 2:**
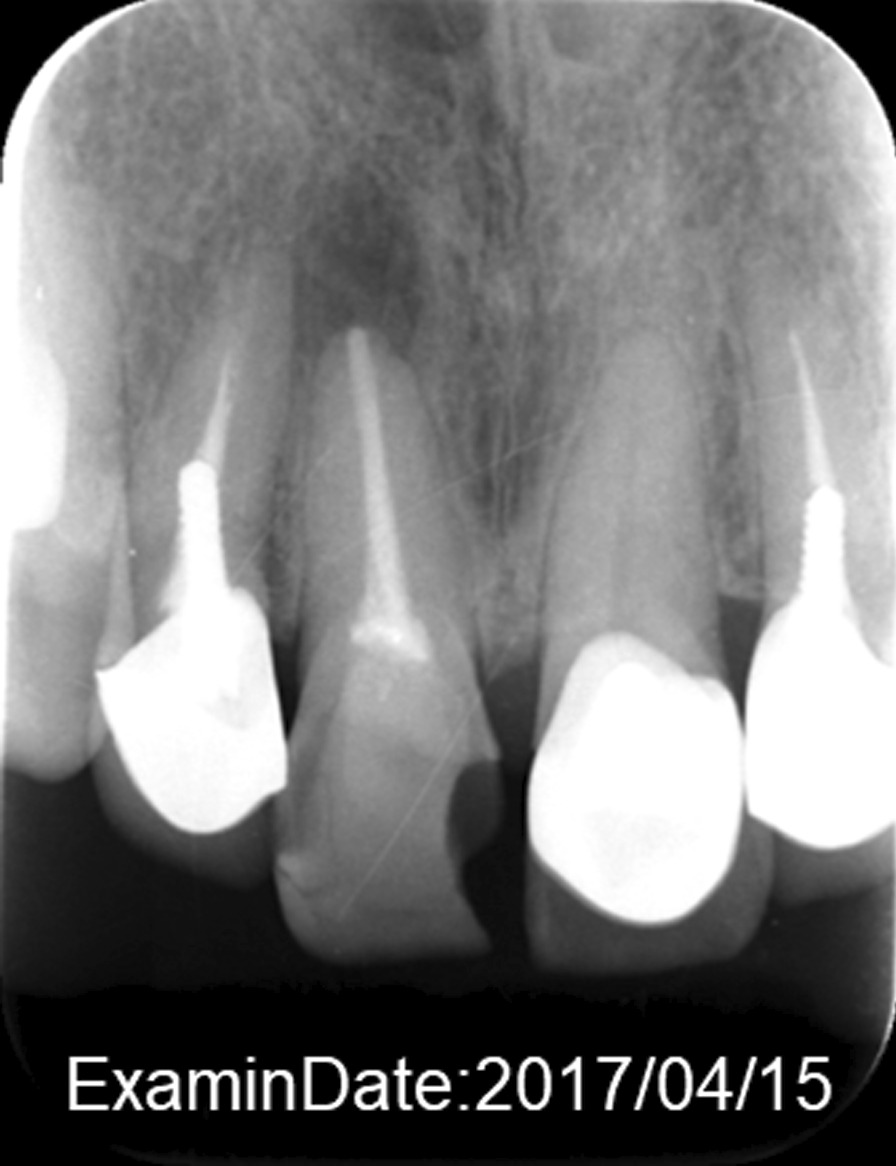
Radiograph of the upper front tooth on 15 April 2017 during the first dental examination. This image shows that the front tooth is missing

**Fig. 3:**
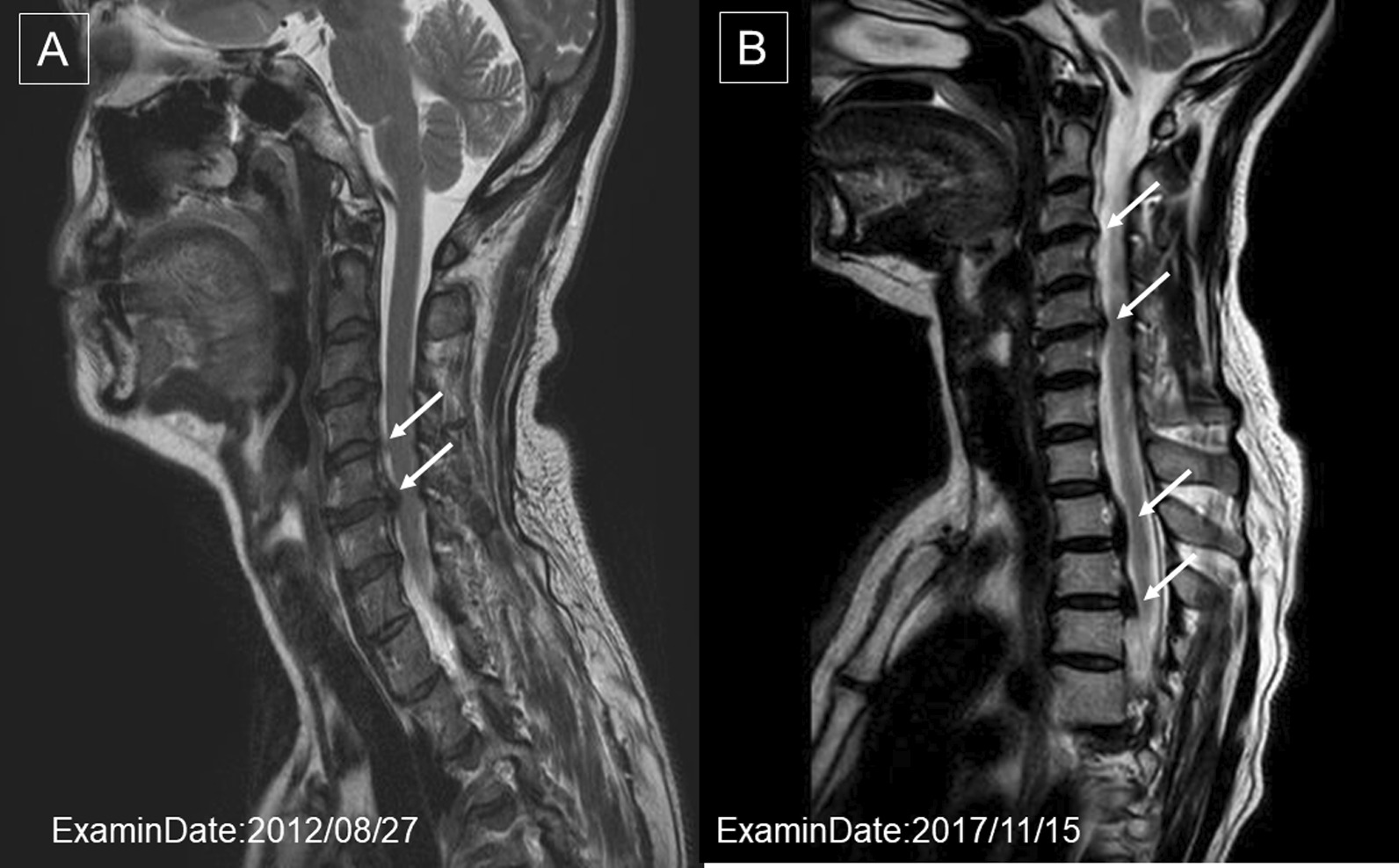
**a** Magnetic resonance image on which the definitive diagnosis of ossification of the posterior longitudinal ligament of the cervical spine on 27 August 2012, was based. The arrows indicate the ossification of the posterior longitudinal ligament of the third cervical spine. **b** Magnetic resonance image of the ossification of the posterior longitudinal ligament of the cervical thoracic spine on 15 November 2017. A new ossification of the posterior longitudinal ligament was confirmed in the fourth thoracic vertebra (arrows)

**Fig. 4:**
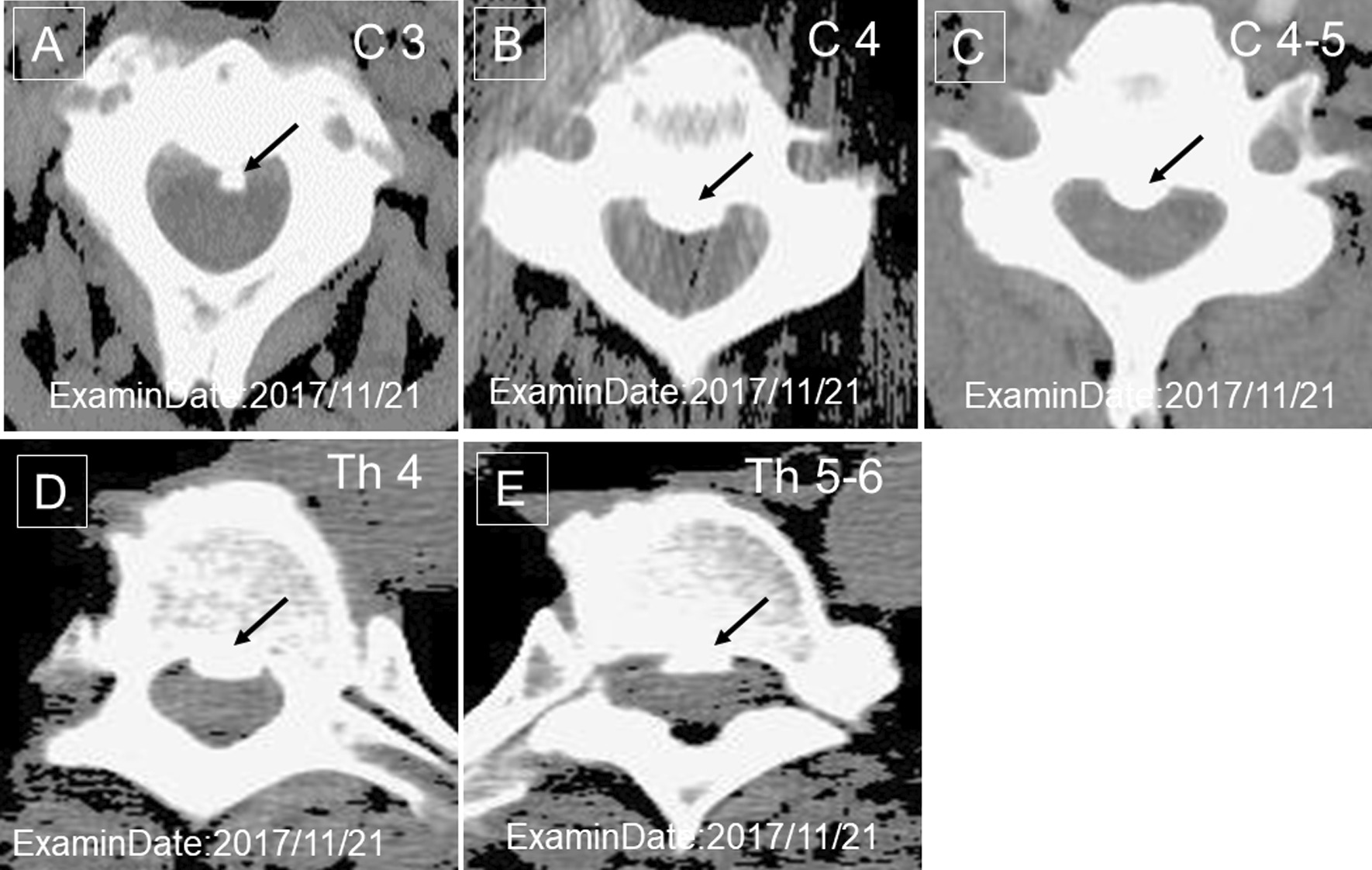
Computed tomography images of the ossification of the posterior longitudinal ligament of the cervical thoracic spine on 21 November 2017. **a** The arrows indicate the ossification of the posterior longitudinal ligament of the third cervical vertebra, **b** the fourth cervical vertebra, **c** the third to the fourth cervical vertebrae, **d** the fourth thoracic vertebra, and **e** the fifth to the sixth thoracic vertebra

**Fig. 5:**
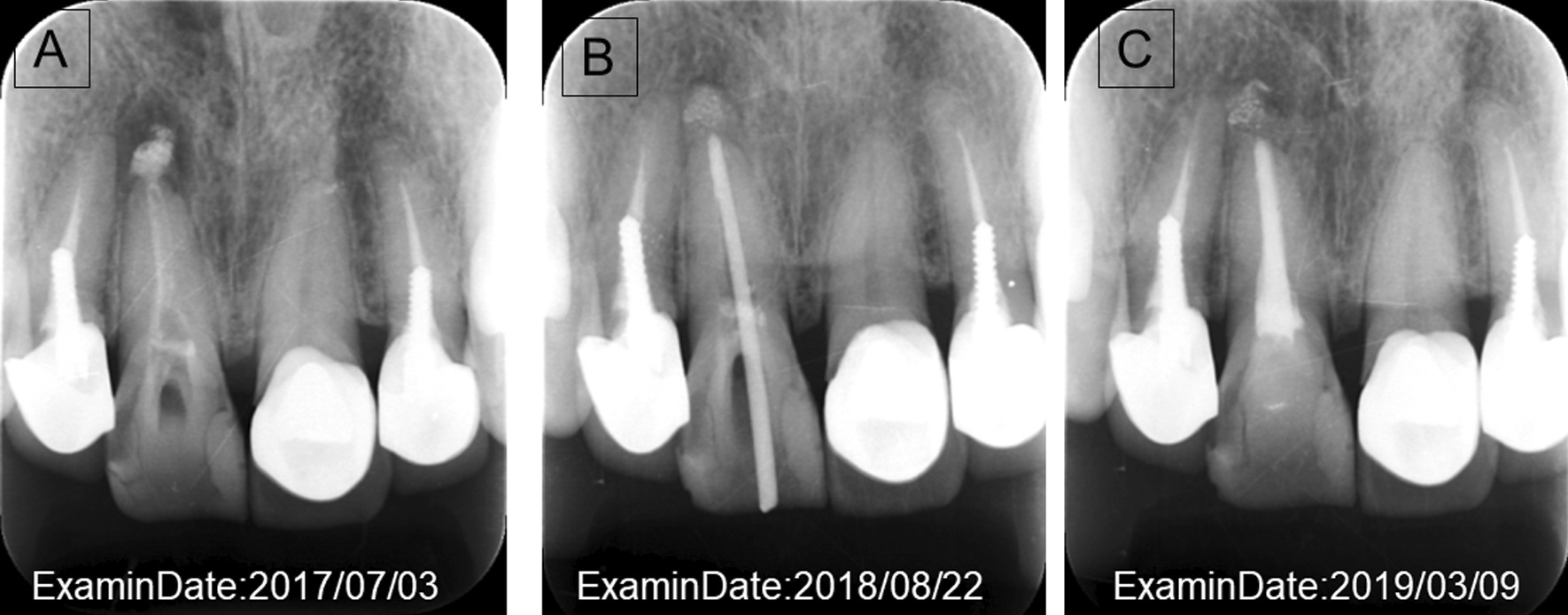
**a** Radiograph during follow-up on 3 July 2017. This image shows that there is a cyst in the tooth root. **b** Radiograph of root canal treatment 22 August 2018. Root canal treatment was initiated; the root canal was thoroughly cleaned and disinfected, then filled with medication and covered; it was closely monitored during follow-up. **c** Radiographs after treatment on 9 March 2019. The root cyst in the affected area improved, and the patient’s pain disappeared

A 41-year-old Japanese woman visited a dentist after a fall and had fractured her upper front tooth (Fig. 2). She had a history of cervical spine contusion due to a car accident in 2006 and was confirmed to have an OPLL of the cervical spine on magnetic resonance imaging (MRI) in August 2012 (Fig. 3a). Furthermore, she was diagnosed with a cervical–thoracic mixed-type OPLL in 2014. We present the MRI (Fig. 3b) and computed tomography (CT) scans (Fig. 4) taken in November 2017 during the study period. She had no remarkable family history of OPLL. She underwent nonsurgical root canal preservation treatment (root canal treatment) for periapical periodontitis, but showed no response to this treatment. Radiography revealed sufficient severity of periapical periodontitis to warrant tooth extraction (Fig. 5a). The patient complained of widespread persistent pain (visual analog scale score of 8) from the sinuses to the head. However, the patient requested nonsurgical periodontal treatment for periapical periodontitis (Fig. 5b).

To treat periapical periodontitis, the endodontist removed the inflamed or infected pulp and carefully cleaned and shaped the inside of the root canal. The extracted tissue of the inflamed pulp was microscopically examined for bacterial presence, and root canal treatment was performed (Fig. 5b) [3]. The space was filled and sealed (Fig. 5c). Thereafter, the dentist placed a crown on the tooth to protect and restore its full function. After restoration, the patient’s front upper tooth continued to function normally. After treatment, the persistent pain caused by periapical periodontitis disappeared and was completely resolved when the patient returned for follow-up in 2019.

## Discussion

In September 2014, cervical and thoracic OPLL were confirmed by a spine specialist on magnetic resonance imaging, and the patient was advised to undergo regular conservative observation. Serum zinc level was measured using atomic absorption spectrophotometry. Blood was collected using a disposable syringe, transferred to a dedicated plastic tube, and incubated under refrigeration. When measuring zinc concentration in serum, plastic test tubes with non-rubber stoppers were used to ensure accurate zinc data.

Before the administration of a zinc supplement, a mineral scan of the skin of the hand was performed in April 2017 [67 μg/mg tissue (normal range 125–155 μg/mg tissue); Oligoscan; Luxometrix-ipc-eu, Windhof, Luxembourg]. The patient was confirmed to have a low zinc concentration and was prescribed a zinc supplement (6–15 mg/day) orally, which was continued until 2019 at the time of periapical periodontitis eradication.

Given the patient’s condition, substantial amounts of zinc were needed before reconstruction of the ligament tissue. In patients with OPLL, other ligament tissues, such as the periodontal ligament, should be monitored. In 2019, when the patient’s periapical periodontitis had completely resolved (Fig. 5c), her serum zinc level remained at 64 mg/dL (normal 80–130 mg/dL). The patient’s serum was zinc deficient despite zinc administration. The results suggested that the patient had multiple ligament disorders and did not have sufficient zinc concentration for ligament tissue regeneration.

In addition, her serum CXCL7 level was 5.1 mg/dL (normal range 7.3–10.3 mg/dL), serum manganese level was < 0.01 mEq/L (normal range 0.6–1.2 mEq/L), serum calcium level was 8.5 mg/dL (normal range 8.6–10.4 mg/dL), serum chromium level was 0.57 μg/dL (normal range < 1.0 μg/dL), serum manganese level was 2.2 mg/dL (normal range 1.8–2.6 mg/dL), and serum selenium level was 14.1 μg/L (normal range 107–171 μg/L). Patients with OPLL generally have high serum glucose levels and a high incidence of combined diabetes mellitus [2]. However, in the present case, the patient’s blood glucose level was normal.

Figures 2 and 5 show the root canal treatment progression during zinc administration in the present case. The patient underwent regular dental examinations. On conservative follow-up by an orthopedic surgeon, the patient did not report any limitations to daily work or life due to OPLL.

## Conclusion

In this report, periapical periodontitis (inflammation of the periodontal ligament) was not initially cured in a patient with OPLL. The patient was in a state of zinc deficiency and began medical treatment with zinc administration. At that time, periapical periodontitis was observed. Approximately 2 years later, the periapical periodontitis was completely cured, and the pain disappeared. To the best of our knowledge, this is the first report to show a relationship between ligament tissue and zinc, and to present therapeutic effects.

The present case suggests that the reduction in the patient’s zinc level, observed in 2019 at the time of periapical periodontitis eradication, was related to zinc absorption in the ligament tissue [1].

Here, we report the association between the PDL and ligament tissue associated with the spinal ligament. We successfully performed nonsurgical root canal preservation treatment (root canal treatment) by administration of zinc for severe periapical periodontitis in a patient with OPLL. The findings of the present case suggest that OPLL was associated with reduced periodontal ligament reconstruction due to zinc deficiency.

Zinc is a typical essential trace element involved in the regulation of various metabolic systems of the living body as a component of metalloenzymes and is important for protein metabolism and genetic information. Approximately 60% of serum zinc is present in combination with albumin, while 30–40% is present in combination with α2-microglobulin [4]. Zinc is considered an important factor that accelerates DNA rotation during tissue regeneration. Zinc deficiency results in reduced synthesis and/or secretion of alkaline phosphatase (a zinc enzyme involved in bone metabolism) and growth factors, such as insulin-like growth factor-1 and transforming growth factor-β. Moreover, osteoporosis is caused by enhanced bone resorption (via osteoclasts) or reduced bone formation (via osteoblasts); in patients with zinc deficiency, bone formation is reduced [5]. Zinc is also a growth-promoting factor and an important parameter for developmental defects and malnutrition [6, 7].

The findings in this case report suggest that it is necessary to test for zinc during the treatment of periapical periodontitis in patients with ligament diseases, such as OPLL.

Ligament tissue is a strong connective tissue that exhibits characteristics that connect the bones. The ability of ligament tissue to regenerate is low, and ligament tissue diseases are difficult to treat. Spinal and periodontal ligament tissues are the most common tissues in the body. Although it is the ligament tissue of another organ, in a patient with ossification of the posterior longitudinal ligament, it was shown that inflammation of the periodontal ligament is also related to zinc as a reconstruction of the ligament tissue. Zinc is an essential trace element. In patients with spinal ligament disorders, compression of the spinal ligament on the spinal nerves is painful, and medical follow-up is required.

## Data Availability

Not applicable.

## Abbreviations

CXCL7: Chemokine (C-X-C motif) ligand 7
OPLL: Ossification of the posterior longitudinal ligament

## References

[1] Tsuru M, Soejima T, Shiba N, *et al*. Proline/arginine-rich end leucine-rich repeat protein converts stem cells to ligament tissue and Zn(II) influences its nuclear expression. Stem Cells Dev. 2013;22(14):2057–70. 10.1089/scd.2012.0695 23442027

[2] Tsuru M, Ono A, Umeyama H, *et al*. Ubiquitin-dependent proteolysis of CXCL7 leads to posterior longitudinal ligament ossification. PLoS ONE. 2018;13(5):e0196204. 10.1371/journal.pone.0196204 PMC5962073 29782494

[3] U.S. Department of Health and Human Services: Oral health in America: a report of the surgeon general. Rockville: Department of Health and Human Services, National Institute of Dental and Craniofacial Research, National Institutes of Health; 2000.

[4] Kodama H, Itakura H, Ohmori H, *et al*. Practice guideline for zinc deficiency 2018. Tokyo: The Japanese Society of Clinical Nutrition; 2018.

[5] Yamaguchi M. Role of nutritional zinc in the prevention of osteoporosis. Mol Cell Biochem. 2010;338(1–2):241–54. 10.1007/s11010-009-0358-0 20035439

[6] Sundaram G, Ramakrishnan T, Parthasarathy H, *et al*. Evaluation of micronutrient (zinc, magnesium, and copper) levels in serum and glycemic status after nonsurgical periodontal therapy in type 2 diabetic patients with chronic periodontitis. Contemp Clin Dent. 2017;8(1):26–32. 10.4103/0976-237X.205036 PMC5426161 28566847

[7] Eda K, Mizuochi T, Iwama I, *et al*. Zinc monotherapy for young children with presymptomatic Wilson disease: a multicenter study in Japan. J Gastroenterol Hepatol. 2018;33(1):264–9. 10.1111/jgh.13812 28452067

